# A313 DISCONNECTED PANCREATIC DUCT SYNDROME: A RETROSPECTIVE OBSERVATIONAL STUDY REVIEWING AN IMPORTANT BUT OVERLOOKED COMPLICATION OF NECROTIZING PANCREATITIS

**DOI:** 10.1093/jcag/gwad061.313

**Published:** 2024-02-14

**Authors:** H Kim, N Ashrafinia, A Fetz, L Tam, D Akhtar, N Aboalfaraj, S Gan

**Affiliations:** The University of British Columbia, Vancouver, BC, Canada; Medicine, University of Saskatchewan, Saskatoon, SK, Canada; The University of British Columbia, Vancouver, BC, Canada; Medicine, University of Saskatchewan, Saskatoon, SK, Canada; The University of British Columbia, Vancouver, BC, Canada; The University of British Columbia, Vancouver, BC, Canada; The University of British Columbia, Vancouver, BC, Canada

## Abstract

**Background:**

Disconnected pancreatic duct syndrome (DPDS) is a complication of acute necrotizing pancreatitis (ANP) characterized by a complete disruption of the main pancreatic duct, leading to the loss of continuity between the pancreatic duct and the gastrointestinal tract (**figure 1**). Due to unopposed leakage of residual pancreatic fluid, patients with DPDS often experience a prolonged disease course with significant morbidity, including complications such as pancreatic fistulas. However, information about the epidemiology, clinical characteristics, and outcomes of DPDS remains scarce.

**Aims:**

To assess clinical outcomes, diagnostic delay, burden of endoscopic and surgical interventions, and complications related to DPDS.

**Methods:**

A retrospective review was conducted for all patients diagnosed with DPDS who underwent either endoscopic ultrasound (EUS) or endoscopic retrograde cholangiopancreatography (ERCP) at our tertiary center between January 2010 and December 2022.

**Results:**

Fifteen patients with DPDS following ANP were identified. The median age of patients at DPDS diagnosis was 56 years (range 33-86 years), with 47% being female. The most common etiology of ANP was biliary (40%), followed by idiopathic (33%) and alcoholic (27%). The median time between ANP diagnosis and DPDS diagnosis was 265 days (range 86-2023 days). Patients underwent a median of 3 (1-9) endoscopic interventions and 1 (0-5) surgical intervention between the initial ANP episode and DPDS diagnosis. 40% of patients experienced intra-abdominal sepsis secondary to infected pancreatic fluid collections. 20% developed pancreatic fistulas (two small bowel fistulas and one cutaneous fistula). 20% developed new-onset diabetes, and 13% had significant worsening of pre-existing diabetes. No mortalities were reported.

**Conclusions:**

There is a significant delay in diagnosing DPDS following ANP, and a substantial burden of endoscopic interventions is required. Frequent complications of DPDS were pancreatic fistulas, recurrent pancreatic fluid collections, and pancreatogenic diabetes. Developing a predictive model for assessing the risk of DPDS development following ANP could aid in earlier diagnosis and potentially reduce morbidity and complications associated with DPDS.

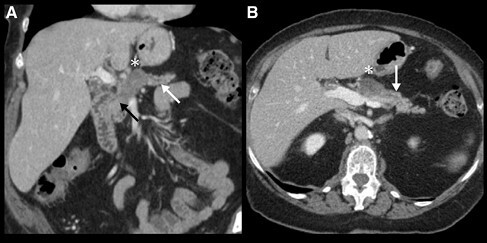

**Figure 1.** Contrast enhanced CT demonstrating typical finding in DPDS in coronal (A) and axial (B) views. CT demonstrates 3cm area of necrosis involving pancreatic neck/body (asterisk) with viable upstream parenchyma in the remainder of the body and tail (white arrow). Pancreatic head/uncinate parenchyma and main duct appear intact (black arrow). B, the distal pancreatic duct is not dilated. *DPDS, disconnected pancreatic duct syndrome.*

**Funding Agencies:**

None

